# Application of some nanoparticles in the field of veterinary medicine

**DOI:** 10.1080/23144599.2019.1691379

**Published:** 2019-12-26

**Authors:** Fady Sayed Youssef, Hossny Awad El-Banna, Hesham Youssef Elzorba, Ahmed Mohamed Galal

**Affiliations:** Pharmacology department, Faculty of Veterinary Medicine, Cairo University, Giza, Egypt

**Keywords:** Nanotechnology, nanoparticles, florfenicol, neomycin, silver, chitosan

## Abstract

Nanotechnology is a fast-growing technology that plays an important great impact on various fields of therapeutic applications. It is capable for solving several problems related to animal health and production. There are different nano-systems such as liposomes, metallic nanoparticles, polymeric micelles, polymeric nanospheres, functionalized fullerenes, carbon nanotubes, dendrimers, polymer-coated nanocrystals and nanoshells. In this review, we mentioned different methods for the preparation and characterization of nanoparticles. This review is concerned mainly on nanoparticle systems for antibiotic delivery which suffer from poor bioavailability and many side effects. Nanoparticles are characterized by many features include their minimal size, colossal surface zone to mass extent. The development of antimicrobials in nanoparticle systems is considered an excellent alternative delivery system for antimicrobials for the treatment of microbial diseases by increasing therapeutic effect and overcoming the side effects. In this paper, we reviewed some antimicrobial nanoparticle preparations and we focused on florfenicol and neomycin nanoparticle preparations as well as chitosan and silver nanoparticles preparations to prepare, characterize and compare their different pharmacological effects.

## 1. Introduction

Nanotechnology is viewed as a quickly developing field that was initially created around 1974 for assembling novel materials ranged from 1 to 100 nm [1–4]. Nano is originated from the Latin word nanus, identical to predominate which implies very little size (1nm equals 10^9^ m) [5–7]. Nanotechnology is viewed as another innovation that takes an interest in numerous fields including science, agriculture and infection treatment delivery assays [8]. Furthermore, nanomaterials have potential impacts for in-vivo and in-vitro biomedical applications and researches [9,10].

Nanoparticles have inventive physicochemical characteristics superior to the mass material due to their enormous surface to volume proportion, higher reactivity, huge surface to volume, stability, bioactivity, bioavailability, controlled particle size, controlled release of drugs, site-specific targeting and controlled arrival of medications [11–17]. Furthermore, nanotechnology has an incredible potential on medication delivery in view of their capacity to enter cells, tissues and organs than macro particles so, conquer poor bio-accessibility and high toxicity of present pharmaceutics [11,18,19]. Medications might be incorporated inside the nanoparticles or connected to their surface [20].

In addition, nanomedicines are portrayed as the utilization of different apparatuses dependent on nanotechnology to widen snappier and additional responses for scientific issues or infection control. It is not handiest in a situation to overcome the difficulties experiencing the customary cure, yet in addition, it allows the comprehension of assorted physiological and obsessive techniques. Profound aptitude for such procedures gives new potential outcomes and remedial norms for effectively current issues [21]. The economy of most nations is reliant on animals. In spite of the rise of numerous illnesses new indicative and helpful tools are created by time to recognize and treat animal sicknesses with the end goal of expanded protein supply for human nourishment [17]. In the field of Veterinary Medicine, nanotechnology has an incredible potential role in the improvement of delivery of the drugs [8]. The curiosity of newly synthetic atoms can provide us a new helpful medicinal drugs with the intention to treat diseases and guard creatures from viral or bacterial diseases and improve wound recuperating. Furthermore, those new mixes could convey drugs into cells for the successful remedy of diseases [21]. Nano-theranostics are considered as a treatment strategy which combine medications with diagnostics, they aim to monitor the treatment response and increase the efficacy and safety of drugs. In addition, they provide a great chance to design and develop such combination agents, allowing the delivery of therapeutics and the detection modality that used before and throughout the treatment regimen [22,23]. Nano pharmaceuticals are a standout amongst the most encouraging and beneficial fields of nanotechnology which has numerous advantages in veterinary medication [24].

## 2. Classification of nanoparticles

There are numerous classifications of the nanoparticles depending on their origin, shape, structure and aim of administration.

### 2.1. According to their structure and their use in veterinary medicine

**Table:** 

Nanoparticle type	Description
*Polymeric nanoparticles:*	There are two sorts of polymers introductory, one is fabricated polymers, e.g. polyethylene glycol (PEG) and the other sort is trademark polymers which their structure rely upon polysaccharides, for instance, inulin and chitosan. Dendrimers are like their structure yet the branches starting from the centre have various measures of spreading focuses this type of nanoparticles are generally utilized in antibody readiness as Newcastle [21].
*Liposomes:*	They were characterized as round biodegradable non-destructive PEGylated particles. Their fluid spotlight passes on water dissolvable medications. Likewise, particles are spreaded by a twofold layer of phospholipid shell and encase the non-water dissolvable remedies. They are moreover might be used to cover various antigens [25]. The outside surface is coated by an external Poly ethylene glycol layer, thus the particles are being protected from being assaulted by the body immune defence. In addition, liposomes can be used for fixing chelated antibodies on their external surface. This type of nanoparticles is in a general sense portrayed by their capacity to pass on both water dissolvable and non-water dissolvable accommodating medicines [26]. Liposomes are very useful for the delivery of both water soluble and water insoluble remedies. They are portrayed by high prosperity in light of their biodegradable structure. Immunoliposomes are another kind of liposome that can connected antibodies called to assault dangerous body cells, they in like manner can be conjugated to outside antigens for immunization purposes [27–29]. Finally, liposomes have a remarkable employment in the delivery of drugs because they are considered as highly biodegradable and biocompatible substances possess high capacity to stack both water soluble and water insoluble simultaneously. The release of the loaded drugs can be done by simple diffusion. In any case, their veritable hindrance is their poor ampleness, short aggregating and speedy arriving of the loaded drugs [30].
*Fullerenes and Bucky tubes:*	Fullerenes are carbon nanoparticles resemble the shape of little ball that able to communicate effectively with pathogens or cells. The buckytubes are round and hollow structures. They can be utilized as either biosensors for different elements identification like immunoglobulins. Because of their needle like structure nanotubes can enter the cells for the disease treatment [30,31,109].
*Microbivores and respirocytes:*	Independently respirocytes take after the elements of red blood cells and white blood cells. While in a well controllable manner they profitably supply the tissues with O_2_ and forgo the amassed CO_2_ using exceptional managerial sensors, pathogens are trapped by microbivores. A change occur to the cleared microorganisms by an enzymatic effect they converted into at first building units which involves amino acids, nucleotides and unsaturated fats [32].
*Nanoshells:*	They are round shaped connected with outer gold layer and used for analysis of malignant tumours by irradiation with Infrared laser.They weaken the force of X-beam and subsequently can be utilized as an adjuvant for radiotherapy. Gold nanoparticles are additionally biocompatible and non-toxic to the body [32,109].
*Quantum dots:*	They are exceptionally little gems from 2 to 10 nm, these molecules possess semi conduction when exposed to light and this feature enables them to be used for optoelectronic purposes [33]. Cadmium, zinc and selenium are the frame of the semiconductor parts [25]. They are composed of a centre and a shell of an inorganic material notwithstanding a fluid covering which can be conjugated to different biomolecules. a crystal is present In the centre. The shade of the discharged light relies upon the precious stone size. They can frame modest and simple long living tests which light as a rule for a considerable length of time or days [109]. Generally Quantum dots can be used for diagnostics and immunodiagnostics purposes [21].
*Solid lipid nanoparticles:*	Balanced out lipids suspended in a fluid arrangement. They comprise of a lipophilic centre which makes them able to be used for cancer treatment). Different hydrophilic medication or antibodies can be conjugated to their outer hydrophilic shell. The outer shell also improves the medication bioprofit capacity. In addition, cationic solid lipid nanoparticles by electrostatic linkage can legitimately tie nucleic acid parts so, empowers their use for quality treatment [28]. Different method of administration can be attained by this type of nanoparticles such as, topical, oral and subcutaneous injection. They are capable to cross the blood brain barrier thus, effectively deliver the medications inside the central nervous system. Other than the strong lipid NPs, the assessment of utilizing fluid lipid Nanoparticles is currently under assessment [21,28].
*Magnetic iron oxide nanoparticles:*	This group is mainly characterized by an external magnetic field that makes them able to be directed via bloodstream to their target cells. They are more suitable for imaging, heat therapy and drug delivery [109]. Their structure comprises a core of iron surrounded by an outer fluorescent layer of silica where the attachment with drugs occurs in which. There is an outer shell which is polymer in nature aid in particles adjustment. Because of their attractive appropriate ties, they are utilized in a few attractive reverberation medicinal applications for disease conclusion and treatment as multi useful theranostic buildings. Polyethylene glycol is used to coat the particles to avoid counteract aggregation of particles and protect them from the immune response. The presence of silica coats facilitates malignancy imaging by improving light retention [28].
*Dendrimer:*	They are hyper branched nanomaterials exceptionally dissolved in aqueous solution, formed from polymers which are extremely little and smaller than the cells of the body [34–36]. Their little size and concoction arrangement maintain a strategic distance from the stimulation of any undesired resistant reactions when infused into the flow [36]. They are looks like a tree of 3D expanded atoms. On the dendrimer surface medications are connected or may be conjugated inside the circle [21]. Through physical and chemical linkage dendrimers are capable to be stacked with many water soluble and water insoluble restorative substances, in addition these medications can be loaded inside the empty centres by means of nonbonding stacking. Covalent conjugation of stacked medications and the dendrimers may happen bringing about the expansion of solidness and improves the remedial productivity. They are utilized fundamentally in cancer treatment. Dendrimers are characterized by their huge, well branched and complicated structure. Medications which are used for imaging can be loaded with dendrimers. Many studies confirmed that dendrimer loaded nanocomposites are very effective antimicrobial agents against *Pseudomonas aeruginosa, E-coli* and *Staphylococcus aureus*. Within the tumour movement is done by the landing of their store of drugs or radioactive compounds. Finally, in case of successful treatment signals are sent with execution of harmful cells. A study were done by a scientific team headed by Landers planned dendritic polymers conjugated with sialic acid that against flu infection,authors reported that the preparation was able to caught infections with inactivation and disposal [30].
*Nano emulsion:*	Nano emulsions have promising restorative incentive as bactericidal and virucidal medications. In the animal body when they interact with bacterial or viral coat by surface tension feature the oil drops stick to the envelope/layer and combine prompting the arrival of the medication within the microbial cells. In addition, nanoemulsion can serve as vehicles for the antigens delivery. In one nanoparticle various antigens can be combined [25]. They are nano sized oily drops in water covered with a thin film of surfactant for physical adjustment of them. Oil in water and water in oil emulsion are the types of nanoemulsion. The ideal stockpiling temperature for nanoemulsions conveyed by low essentialness methodologies are 4°C and room temperature and the capacity time is over 2months [34]. The water/oil nanoparticle emulsions are able to release the antigens consistently so they act as adjuvants for improving the arrival of antibodies with in high titres [35]. When they are given systemically a few analysts alert the symptoms of fat drops on RBCs and sperm cells [36]. Numerous audits considered nanoemulsions safe for use without inconveniences on cells of eukaryotes [37–40].
*Nano bubbles:*	At room temperature, they remain stable however they accumulated together to form micro-bubbles when they are marginally warmed when presented to ultrasonic waves. They are mainly used in drugs delivery especially delivery of drugs into the cancer tissues. Liposomal nano bubbles are likewise utilized in gene therapy [37].
*Aluminosilicate nanoparticles:*	They are (short-chain polyphosphate coupled with silica nanoparticles) that used to accelerate natural clotting mechanism so, leads to reduction of bleeding [38].
*Polymeric micelles:*	They are composed of a core of hydrophobic property that facilitates the transportation of hydrophobic drugs. The hydrophobic core is coated by a water soluble coat making them highly water soluble.They are also mentioned that they were made with amphiphilic polymers such as (ecaprolactone) or PLGA. Generally used for focused drug transport; supply drugs which are less water soluble which includes paclitaxel and amphotericin B [50–56].
*Polymer coated Nano crystals:*	They prevent aggregation and facilitates in organizing a strong nanosupension Macrophage dependent delivery to sites of HIV infection and sequestration [56].
*Polymeric Nano spheres:*	Uniform round frameworks substantially their size is smaller than a micron produced using polymers which are either biodegradable or non-biodegradable polymers. Ground-breaking for transdermal medication conveyance, it might be used in the analysis of type 2 human epidermal advancement factor receptor and in-vitro integrin cancer cell growth [41].
*Metallic nanoparticles:*	There are different types of metals used in the nanosystem one of them is gold that is mainly utilized in cancer therapy. Silver, manganese and platinum nanoparticles are another type of metallic nanoparticles, each of them possess a metallic centre that coated by a protected layer. In addition, various antibodies and chelated radionuclide are loaded on metallic nanoparticles. Particles are attached to polyethylene glycol which protects them from the invulnerable immunological this is done in order to maintain a strategic distance from non-specific binding [42–46]. Some metallic nanoparticles especially bimetallic nanoparticles were utilized for disease treatment including silver/gold nanoparticles [47], silver–selenium [48], or gold-platinum [49].

### 2.2. According to their origin

According to their origin, they are classified into organic, inorganic and hybrid nanoparticles.

#### 2.2.1. Inorganic nanomaterials

Generally, they are save and are typically biocompatible and are less cytotoxic. They have novel electrical and optical characteristics which can be adjusted during assembling. This group includes different inorganic materials, for example, gold, silver, iron oxide and calcium phosphate [50].

#### 2.2.2. Organic nanomaterials

##### 2.2.2.1. Proteins and peptide nanoparticles

They are utilized for the most part in quality delivery measures (e.g. gelatin) because of their low lethality, minimal effort of creation, high biodegradability and bio similarity. They can likewise cooperate with different payloads. There are two sorts of utilized gelatins type A (extracted from tissues by acids) and type B (separated by bases) which vary in their isoelectric focuses [51,52].

The utilization of gelatin B offers an incredible bit of leeway as its isoelectric point is 4.8–5.2, which enables it to interface with positively charged atoms at physiological pH. Gelatin B is contrarily charged; in any case, when it experiences an endosome, it changes its charge and become decidedly charged, with the goal that the emphatically charged burden will be easily discharged inside the phones. For their utilization to convey DNA, the gelatin molecule must be conjugated to an exceptionally positive sub-position (for example, Protamine sulphate) to trap the DNA inside the atom complex [53].

The hereditary material can likewise be conveyed together with helpful operators simultaneously when both are co-stacked in centre shell NPs in which the centre protein is made of egg whites [54].

#### 2.2.3. Hybrid nanoparticles

They are composed of more than one sector of nanoparticle, e.g. mixture of polymeric NP and liposomes that results in a polymer-lipid hybrid system. The formed compound is characterized by a core of biodegradable hydrophobic polymers loaded with hydrophilic drugs for simultaneous release of the drugs. The lipid layer controls the water penetration to the nanoparticles and drug release from them [55,56].

### 2.3. According to their shape

They are classified into (spheres, tubes or liquid drops), depending on the aim of application (e.g. therapeutic, diagnostic, vaccine administration, nutritional) [56].

### 2.4. Miscellaneous classification

1. Immune-invigorating edifices: they are considered as supramolecular saponin adjuvant particles. Their main use is to trap different viral antigens via hydrophobic combination with viral wrap proteins [57,58].
2. Virus-like particles: Particles of self-collecting property going in size from 20 nm to 800 nm. Due to the lake of the nucleic corrosive, in this manner, they can invigorate strong insusceptible reaction without having the option to actuate contamination [59].
3. Self-gathering frameworks/proteins: They drive more elevated amounts of protein quaternary structures to be utilized in human and animal vaccination [60].

## 3. Different methods for preparation & characterization of some nanoparticles

### 3.1. Different methods for preparation of nanoparticles

**Table:** 

Method	Description
A.Emulsion-Solvent Evaporation Method:	It entails 2 steps. This method is modified into emulsification with high pressure and another method known as solvent evaporation [61].
B. Twofold Emulsion and Dissipation Technique:	This method based on encapsulation of water soluble drugs through mixing of organic polymer solutions with hydrophilic drug solution this step is followed by stirring powerfully to form water/oil emulsions [62].
C. Salting Out Technique:	Basically depend on isolation of a solvent of water miscible property from watery solution through a salting out impact [63].
D. Emulsions Diffusion Method:	It relies upon dissolving the polymer in part in a water-miscible dissolvable and completely immersed with water. In the long run shaped an emulsion in a watery arrangement that contains a stabilizer, prompting dissolvable dissemination to outside stage and development of nano spheres or Nano capsules. Detriments are exorbitant volumes of water to be disposed of, bringing down exemplification execution [64].
E. Solvent Displacement/Precipitation method:	Includes precipitation of a preformed polymer from a characteristic arrangement and dispersion of normal dissolvable in a fluid medium inside the nearness or non-attendance of surfactant. Medication is disintegrated in a semi-polar water dissolvable including CH3)2CO or ethanol. This strategy is proper for ineffectively dissolvable medications [65].

### 3.2. Different methods for nanoparticles characterization

#### 3.2.1. Molecule size of nanoparticles

Particles size influence the medication discharge. Little particles give the bigger surface area. As a final product, the limit of the medication stacked onto them can be revealed to the molecule surface so speed medication discharge [65].

**There are several tools for determining nanoparticle size:**

#### 3.2.1.1. SEM

Scanning electron microscope is giving a morphological assessment with direct perception. They give bound data about the size dispersion and the normal of genuine populace. This technique is tedious, costly and frequently need reciprocal insights about estimating dispersion [66].

#### 3.2.1.2. TEM

Transmission electron microscope is mainly utilized for recognizable proof of the morphology of the prepared nanoparticles.

#### 3.2.1.3. Atomic force microscopy

2D and 3D AFM images are used to determine surface topography and roughness profiles of nanoparticles.

#### 3.2.1.4. Zeta potential

Used for determine zeta potential value, size of nanoparticles and surface charge [67].

#### 3.2.1.5. Surface hydrophobicity

Determined by numerous techniques along with hydrophobic interplay chromatography, biphasic partitioning and adsorption of probes [68].

#### 3.2.1.6. Drug release

Drug release assays also are just like drug loading assay which is classed to investigate the drug release mechanism [69].

**Summary of several tools for determining nanoparticle size:**

**Figure:**
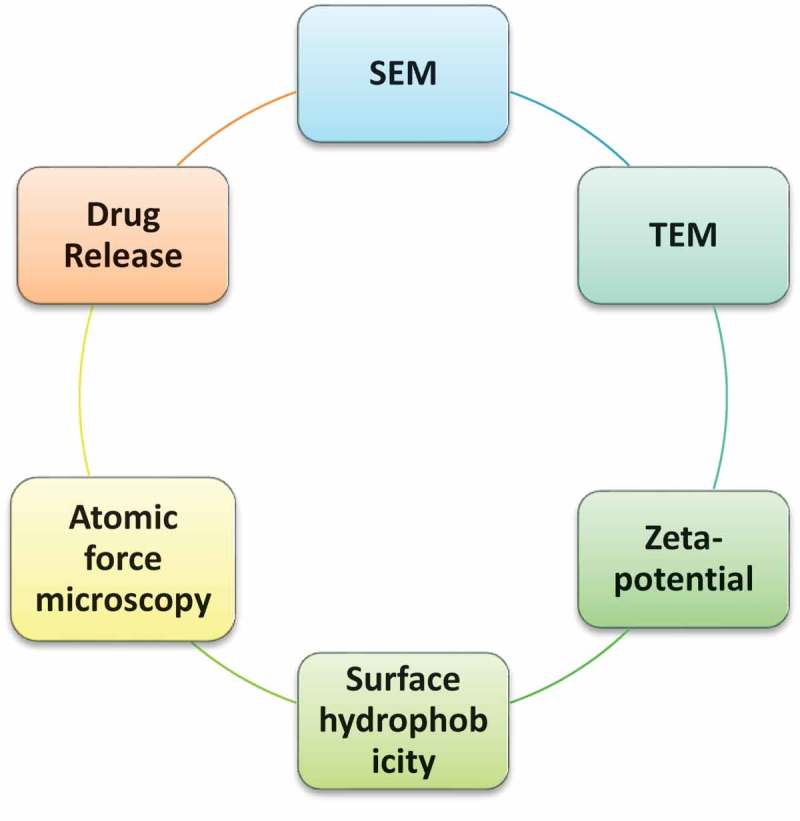

## 4. Applications of nanotechnology

Nanotechnology is viewed as an incredible solution for numerous issues especially for health of animals and improvement of their production.

**Figure:**
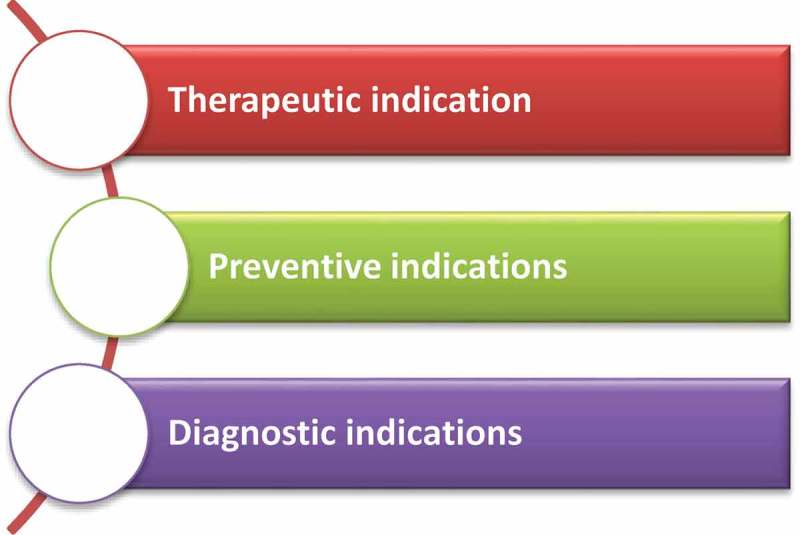

### 4.1. Possible applications of nanotechnology in medical science

**Table:** 

***4.1.A. Therapeutic indications:***	1. It is easy to control their physical/chemical features over the span of assembling in accordance with the purposeful application which gives an unending assortment of releases. Thus empowers personalization of the healing and diagnostic concept **[71]**.
	2. They permit the assembling of definitions that contain every helpful and demonstrative operator in a solitary framework **[23]**.
	3. Their use enables payloads in excessive quantities due to large ratio between surface area and extent **[72, 73].**
	4. Nanoparticles are described by their dependability even under exorbitant weight and temperature **[74]**.
	5. They are able to penetrate blood brain barrier due to their small size, further they can reach to their target sites of action effectively and avoid their detection and elimination **[52].**
	6. They can easily consolidate with the natural contraption of the organism that prompts terrible immunological responses **[30].**
	7. Enables various preparation administration by utilizing capsules that used by injection with local utility **[75].**
	8. Permit constant following of cure/diagnostics **[34].**
	9. They are ready to control medication discharge. They target neurotic sores effectively, specifically and gather inside them prompting extra proficient treatment, improvement of medication bioavailability, decline the required treatment portion which has a monetary effect **[30].**
	10. Provide sustained release of different antibiotics, vitamins and hormones **[76].**
	11. Permit cure of intracellular pathogens like brucella and leishmania further, multi antibiotic resistant pathogens such as MRSA **[77, 78]**.
	12. Nanoparticles can be utilized for malignancy cell disposal this accomplished by method for vehicle of chemotherapeutic operators, warming the cells, particular immunological ambush of the cells **[48].**
	13. The utilization of nanoparticles with tumor-exact antibodies permits the end of metastatic malignant growth cells far away from the first injuries **[53].**
	14. Different substances like polymers, lipids and metals can be used for nanoparticles formation; they have a wide extent of dynamic parts, like chemotherapeutics and quieting medications **[79]**, separate authorities for imaging and discovering **[49]**, nucleic acids, proteins **[50]**, in addition antimicrobials. A wide extent of microbial infections including tuberculosis can be treated by nanostructured antimicrobials **[49]**. The usage of nanocarriers is viewed as one of the valuable huge pieces of nano medicine this was confirmed by many studies that revealed the effect of them in treatment of many disease depending on traditional pharmacological bases **[80 - 82]**.
	15. The improvement of chemotherapeutic operator which is epitomized in the nanoparticle is increasingly compelling for the treatment of constant illnesses with significantly less harmfulness and cell obstruction contrasted with standard chemotherapy specialists. For example, are very powerful inside the remedy of ovarian carcinomas while used together, but due to their excessive toxicity, lipid nanoparticle preparation of doxorubicin and paclitaxel active towards most cancer resistant cellular lines validated the efficacy of nanoparticles in tumor growth control with little toxicity **[48]**.
***4.1.B. Preventive indications***:	1. Gives new plans to the headway of new antibodies with increasingly secure stable adjuvants **[36]**.
	2. Numerous preliminaries brought about the development of remote sensors administrated to the patients under their skin in order to measure certain target proteins level **[48]**.
***4.1.C. Diagnostic indications:***	1. Incorporation of nanoparticles with tumor-particular antibodies enables early prognosis of most cancers which considers better endurance rates and checking of the total casing for metastatic injuries **[53].**
	2. As imaging operators, which possess long duration of action and allow imaging substance to be used in a repetitive manner without side effect on liver or kidney **[31].**
	3. Nanorobotics can be utilized in investigatory and mending miniaturized scale medical procedures. Additionally, they can convey nano cameras for constant aid surgeries **[21, 33]**.
	4. It provides an extremely fast screening and diagnostic property. They are able to detect large number of genes and proteins because of the presence of the usage of nano array chips with high-density **[32]**.

### 4.2. Uses of nanotechnology in veterinary medicine

Nanotechnology has a significant effect for veterinary prescription in numerous fields incorporates therapeutics, diagnostics, tissue building, immunization generation and disinfectants. The Nano-applications are as of now being used inside the region of creature wellbeing, rearing, proliferation and sustenance of creature. The vehicle of the medicine straightforwardly into the target cells empowers utilizing exceptionally low dosages that progressively diminish the medication build-ups and withdrawal period in homestead animals [56].

### 4.3. Potential uses of nanotechnology in animal production

A couple of potential uses of nanotechnology in animal production includes; the executives of drug, vitamins, probiotics and nutritional supplements, similarly, using nanoparticles for detection and removal of causes of infection without surgery. However, the normal use of antibiotics in animal production can leave a residue that affects the final consumer whilst even as using nanotechnology reduces the range of antibiotics used because of their Nano size [81].

This was confirmed by Fattal et al. [82] who studied the impact of ampicillin loaded to nanoparticles on mice infected with *Salmonella typhimurium*, and result confirmed that mice treated ampicillin joined to nanoparticles confirmed higher survival ratio much like ordinary manage group, the distinction becomes because of the earlier required 40 instances less antibiotic to reap the identical impact with a higher distribution in tissues, so tested the lower amount of antibiotic required to present the same effects. Animal production, need to gain from this period thru the usage of nano-fertilizers that deliver vitamins to particular places within the forages, inside the required quantities. This may be completed with the useful resource of magnets. Similarly, nanomaterials may be utilized in combination with hydrogels or zeolites to enhance water excellent with the aid of using soaking up poisonous substances [83].

### 4.4. Impact of nanotechnology for animal health and nutrition

Nano minerals are inexpensive, utilized in lower quantities, and act as growth-promoting and immune-stimulating agents so, produce many benefits for animal feed production. Similarly, they also can help to manipulate feed pathogens and improve fermentation process in the rumen. Nano zinc oxide is considered one of the promising Nano minerals that used to improve the growth rate, immune response and also disorders affect livestock reproduction. Further diarrhoea in young piglets can be reduced by Nano zinc [84]. Nano zinc decreases the somatic cell count in dairy cows suffered from subclinical mastitis [85].

Microencapsulation of feed components is done in order to protect them from oxidation and break down by light and oxidation, further it keeps them away from the lysis by enzymes of the digestive system such as proteases, also promote stability at various values of pH, with better dispersion and good mixing of lipophilic additives, and will prolong their action. A big problem affecting animals and human is the mycotoxicosis present in about. They may be detected in about 25% in the feed of animals. Nano silica and nano magnesium oxide are considered a powerful nano antimycotoxin that bind successfully to aflatoxins and inactivate them [86].

### 4.5. Application of nanotechnology in breeding and reproduction

Diagnosis and treatment of reproductive problems, oestrus detection, sperm freezing and the direct calving interference are done by several nanotechnology applications. Further, many reproductive problems like retained placenta can be treated by nanotechnology. In addition, nanoparticles have a great impact in protection and sustaining the release of hormones of reproduction like steroid hormones or gonadotropic hormones [87].

One of the most up to date and powerful apparatus utilized in creature generation is nanotubes they are utilized because of the issue of surprising expense and longer time needed for rearing and proliferation, a nanotube might be established beneath the pores and skin to offer precise time measurement of modifications in oestradiol organize within the blood. Oestrus in animals can be discovered by nanotubes since their ability at the time of oestrus to bind on the oestradiol antibody via fluorescence of infrared. Further, nowadays Micro fluidics facilitates in vitro fertilization processes [88].

Nano sensors are another device with a cell probe. These probes are mainly utilized in diagnoses of genital tract infectious illnesses, metabolic and hormonal issues or may be the detection of oestrus [89].

For animal, sterilization nanoparticles can be used as contraceptives depending on the toxicity of some metallic nanoparticles which includes cadmium whilst given in low doses. Course of metallic nanoparticles to the reproductive tract of the animals increases their impact there [56].

## 5. Nanotechnology applications in veterinary therapeutics

Nowadays, nanotechnology is supposed to play a promising role in veterinary therapeutics [90]. One example is that the mixture nanocarrier-mediated therapy is effective in treatment of diseases affecting animals producing food for human consumption food [91]. From current reviews, it is far advised that quantum dots (QD) are recommended to be used for *in vivo* imaging in small animal [92]. In addition, nanotechnology is presently carried out for the treatment of trypanosome. This appeared in the facilitated delivery of diminazene (DMZ) to the target site of action. The porous cationic nanoparticles used stepped forward the potential focused on of trypanosomes. Evaluation of medical parameters after nanoparticle treatment revealed a partial reduction of allergic conditions [93].

### 5.1. Nano vaccines and nano adjuvants

Nanoparticles are more and more used in the field of Veterinary vaccine production due to their ability to improve immunological responses. In addition, they are able to serve as adjuvant in order to make the antigens slowly released this will increase vaccine performance [94]. The use of nanoparticles for loading of antigens results in targeting the lymph nodes leading to vaccine performance improvement [95].

#### 5.1.1. Examples explain the various types of nano vaccines used in veterinary medicine

A. Examples of nano emulsion vaccines are recombinant B. anthracis spore-based vaccine and influenza vaccine. B. Following oral administration of vaccine loaded on PLGA nanoparticles, they produce Immunoglobulin type G and Immunoglobulin type A immune reaction example of these vaccines are *Helicobacter pylori*, Tetanus toxoid, *Bordetella pertussis* vaccine and Bovine para influenza type III vaccine. C. Recombinant Leishmania SOD vaccine is an example of vaccines that loaded on chitosan nanoparticles and given by subcutaneous injection, in addition, TB vaccine loaded on chitosan is given through respiratory tract, also pneumococcal antigen a vaccine and Streptococci equi vaccine are loaded on chitosan and given by intranasal route. Gold nanoparticle-based vaccine is given in opposition to foot and mouth disease. D. Empty capsid and centre like particle vaccines of the virus which affect horse and called African horse sickness [96–107].

## 6. Implementation of nanotechnology in pet animal care

For pet animal care nanotechnology was likewise connected to grow new items. They are used in the improvement of surface freshening up and disinfectants due to their physicochemical properties. For example, silver nanoparticle is incorporated in shampoos for topical use [90].

## 7. Current limitation and safety of nanoparticles

In general, most nanoparticles are safe, but a few may additionally have risky outcomes such as; extended pulmonary publicity to carbon nanotubes might lead to reproductive problems to the workers of the pharmaceutical organizations [108]. Furthermore, the development of attractive nanoparticles made from iron oxide inside the edge, or through harms expedited because of unsteady authoritative between the medication and the particles that moreover may dispatch the medication in solid tissues rather than the objective tissues. The incomplete arrival of the direction away from its objective tissue or organ will now cause healthy tissue toxicity as well as the conveying of dosages in a sub-therapeutic level at the objective component. Their ability to move various organic restrictions inside the casing, for instance, the blood–brain barrier commits any error has extraordinary outcomes, evenly at the environment, for example, the expanding call for radionuclides, also nano fibres with carbon likewise are implicated to consume the ozone layer in the biological system [21,108–110].

## 8. Application of nanoparticles in drug delivery systems

Nanoparticles in the field of pharmacology are considered an ideal drug delivery system that not only guard animals from viral or bacterial infections but further enhance wound healing and can reduce pain. Additionally, those new compounds deliver drugs to the target tissues and organs. Those frameworks can have an impact at the pace of assimilation, appropriation, digestion and discharge of medications or different substances in the body and permitting the observing of the drug dynamics, acquire a therapeutic effect, ensure bioavailability, stability, lengthen the length of movement, reduce the frequency of doses required to preserve the therapeutic responses and reduce the toxicity [108].

### 8.1. Classification of nanoparticle delivery systems

From the delivery system perspective nanoparticles are assembled into Polymer-based, lipid-based and metal-based delivery systems:

*Polymer-based delivery system* such as dendrimers, nanospheres, niosomes and polymeric micelles. Also, it was classified into Natural polymers such as chitosan, collagen, gelatin and engineered polymers like PLA and PLGA. *Lipid-bases delivery system* such as nanoliposomes, solid lipid nanoparticles and lipid vesicles. *Metal-based delivery system* such as nanotubes, metal colloids, gold nanoshells and fullerenes [111].

## 9. Disadvantages of use of the native antimicrobials

The invention of antibiotics inside the twentieth century resulted in reduction of the morbidity and mortality caused by microbial infections. In spite of the great progress with antimicrobials improvement, many infectious diseases, especially intracellular infections, stay difficult to be treated. In continual infections, micro-organisms stay alive from months to years. Insufficient and occasional shipping of antimicrobial agents may also cause inadequate therapeutic index with the improvement of antibiotic resistance, neighbourhood and systemic facet effects like nausea, vomiting, irritation, scaling and gut microflora reduction are taken into consideration terrific problem that encourages the pharmaceutical agencies and researchers to simultaneously increase novel antibacterial capsules and delivery system to overcome multidrug-resistant microorganism [112,113].

## 10. Favourable circumstances of utilizing nanoparticles as another drug delivery system for antimicrobial therapy

They upgrade remedial viability and symptoms of medications. They improves the dissolvability of inadequately water-solvent medications.

Nanoparticles have distinctive properties such as ultra-little, controllable size, enormous surface zone and high reactivity thus, facilitate administration of antimicrobial medication. They conjointly enhance therapeutic effectiveness and minimize limits undesirable aspects of pharmaceutical compounds. In addition, they potentiate the solubility of less water-soluble drugs. Further, they raise the potency of therapeutic compounds and increase the specificity towards target cell or tissue. They have incredible favourable circumstances such as their ability to Prolong the systemic circulation lifetime of drugs, improve bioavailability, decrease sedate digestion and empower an increasingly controllable arrival of helpful mixes and the conveyance of at least two medications at the same time for mix treatment. They also are used for Renewing of old pharmaceutical bases which were continued used. They are capable to reduce toxicity and collateral effects of conventional pharmaceutical compounds. In addition, they can overcome the hazards of drug residues as they produce low residues in animal products. One of the important advantages of using nanoparticles is the reduction of the needed doses, frequency of dosage and concentration of drug during the course of treatment [114–116].

## 11. Review of some antimicrobial nanoparticles used in veterinary medicine with special consideration to florfenicol and neomycin

Covalent connection of a penicillin polyacrylate nanoparticle with roughly 100 nm was reported to be effective in treatment of MRSA, through protection against action of bacterial β-lactamases [117]. Ceftiofur-loaded PHBV Poly (3-hydroxybutyrate-co-3-hydroxyvalerate) was reported to improve the infectious diseases treatment in the livestock [118]. Oral administration of tilmicosin-loaded lipid nanoparticles in broiler chickens was founded to be have more bioavailability and suggested to be an efficient delivery system for tilmicosin [119]. Streptomycin coated chitosan-magnetic nanoparticles by incorporation method showed fast release profiles, slower as time progressed and have more enhanced antibacterial activity against methicillin-resistant *Staphylococcus aureus* [120].

### 11.1. Florfenicol

Florfenicol is a fluorinated analogue of thiamphenicol and considered as an economical broad-spectrum antibiotic which is widely used for prevention and treatment of many against gram-positive and gram-negative bacterial infections [121]. It has been widely used to prevent and treat the respiratory diseases in bovine [122], vibriosis in fish [123] and swine and chicken diseases in the People’s Republic of China since 1999 [124]. Its mechanism of action is similar to that of thiamphenicol and chloramphenicol. Florfenicol overcomes the risk of chloramphenicol-related aplastic anaemia and abolishes the bacterial acetyltransferase-mediated drug resistance due to modified chemical structure [125]. Florfenicol has high efficacy at lower concentrations than its structural analogues against many chloramphenicol-resistant or thiamphenicol-resistant strains [126].

There are some disadvantages of florfenicol, firstly its poor solubility in aqueous solutions [126] so organic solvents are normally utilized in its clinical fluid definitions. The elimination half-time of florfenicol is less than 3 h in some animals [127–129]. What is more, frequent dose is required in clinical applications to acquire better helpful efficacies. Administration in a frequent manner would build work cost and creature stress. In this way, advancement of florfenicol novel plans has promising viable qualities.

#### 11.1.1. Review of some florfenicol nanoparticle preparations

The following studies demonstrated different nanoparticle preparations for florfenicol:

##### 11.1.1.1. Study on florfenicol-loaded solid lipid nanoparticle suspension

Preparation was done by hot homogenization and ultrasonic technique. Value of zeta potential was >47 mV. Minimum inhibitory concentration was 6 and 3 μg per mL against S. aureus and E.coli, respectively. Median lethal dose was >5000 mg per kilogram body weight [129].

##### 11.1.1.2. Control release of florfenicol using silica nanoparticles as a carrier

Florfenicol was loaded on silica nanoparticles as a carrier in aqueous solution. Through a natural cooling process from 95°C to room temperature adsorbtion of florfenicol on silica nanoparticles in aqueous solution was obtained. Characterization was done by transmission electron microscope, Zeta sizer laser particle size analyser, FTIR, thermal gravimetric analysis. Results revealed that the adsorption was done without degradation. Florfenicol nanoparticles showed a slower sustained release of florfenicol than the rapid release of native florfenicol [130].

### 11.2. Neomycin

Broad-spectrum poorly absorbed bactericidal aminoglycoside antibiotic with and indicated for prevention and treatment of bacterial enteritis in poultry occur due to caused by bacteria susceptible to salmonellosis and colibacillosis. Its mode of action is at the ribosomal level. When administered orally, only a fraction (<5%) is absorbed systemically when administrated orally. Neomycin sulphate was effective at 11 or 22 mg per kilogram body weight, when administered in the drinking water for 5 days. Neomycin disadvantages are nephrotoxicity and ototoxicity [131].

#### 11.2.1. Review of some neomycin nanoparticle formulations

Neomycin loaded chitosan nanoparticles was formulated and evaluated and studied for its antimicrobial activity. The prepared formulation showed entrapment efficiency (%EE) of65.5%, scanning electron microscopy round shape and smooth surface. The in-vitro release profile was found to be 96.65% sustained up to 330 min. In addition, the drug release enhanced when compared to pure drug [132].

A comparison of different neomycin nano biocomposites marketed ointment was done for in-vitro and in-vivo evaluations. Authors concluded that the prepared zinc chitosan neomycin nanoparticle (0.2% neomycin) was found to be the best formulation of neomycin containing less than half of the concentration of neomycin of nemozin ointment (0.5%) with low side effects and high efficacy at low doses [133].

Neomycin loaded silver nanoparticle preparation was found to have a synergistic effect in 45% of mastitis-inflicting Staphylococcus aureus in milk samples. Authors proposed that the synergistic impact might be because of drug transport of silver nanoparticle to the cell. It is far well known that hydrophobic groups are discovered in cell membranes. Gentamicin and neomycin are hydrophilic however silver nanoparticles are hydrophobic. For this reason, silver nanoparticle in contrast to antibiotics can easily bypass cellular membrane and reach to the target cell effectively [134].

## 12. Polymeric nanoparticles with special interest to chitosan

They are classified into two types polymeric nanoparticles natural and synthetic. Examples of natural polymers are chitosan which is one of the natural polymers are inulin and chitosan which are polysaccharide in nature.

### 12.1. Chitosan nanoparticles

Chitosan is a promising nano-carrier to release the encapsulated drug to the targeted tissues [135].

#### 12.1.1. Preparation of chitosan nanoparticles

There are different methods for synthesis of Based on ionic gelation method of TPP with chitosan [136].

#### 12.1.2. Properties of chitosan nanoparticles

Preparation is done by the interaction of oppositely charged macromolecules [137]. Compared with other biological polymers, chitosan is one of the most extensively used polymers due to biocompatibility, biodegradability and less toxicity. The interaction can be controlled by the charge density of Tripolyphosphate and chitosan. They are considered as ideal drug delivery system for poorly soluble, poorly absorbed substances [138].

#### 12.1.3. Review for antimicrobial activity of chitosan nanoparticle

Ampicillin-loaded chitosan nanoparticles were studied for their antimicrobial activity against Escherichia coli. The study revealed that chitosan nanoparticles capably prolonged the ampicillin delivery due to nanoparticle size and increased surface charge, resulting in inhibition of *E. coli* growth [139].

A Generation protocol for the synthesis of chitosan nanoparticles loaded with florfenicol through the ionic gelation method was studied. In this way, the synthesis of chitosan nanoparticles loaded with florfenicol could provide advantages when it comes to protecting, transporting and releasing the drug in fish of economic interest in a controlled manner. The results obtained with FTIR spectrometry corroborate the formation of the Q-TPP nanoparticles with the changes observed in the infrared spectra patterns between the matrix chitosan and the synthesized nanoparticles. Q-TPP nanoparticles can carry between 48% and 50% of florfenicol inside. The in vitro release assay of the antibiotic showed a controlled and stable release over time under conditions of acid pH and controlled temperature of 15^°^C for up to 10 days [140].

## 13. Metallic nanoparticles

### 13.1. Silver nanoparticles and their anti-microbial activity

Different methods are involved in synthesis of silver nanoparticles one of them is the precipitation method by using a reducing agent trisodium citrate (TSC) [141].

Silver nanostructures were reported to have a potential effect on gram-positive like *S. aureus* and gram-negative bacteria *Echerichia coli, Klebsiella and Pseudomonas*. These nanoparticles act by attacking the respiratory chain and cell division, finally leading to cell death. In addition, the STEM (Scanning Transmission Electron Microscopy) confirms the presence of silver in the cell membrane and inside bacteria [142]. Previous studies demonstrated that silver nanoparticle have many different pathways of bactericidal activity either by the effect of release of free radicals or reactive oxygen species (ROS) in addition the interaction of silver nanoparticles with bacterial cells in the form of connection with the respiratory enzymes or diminishing the intracellular ATP levels may lead to breaking down the cell wall of the bacteria [143,144]. The effects of silver nanoparticles on *Staphylococcus aureus* contaminated open wounds healing in mice was studied and results showed a remarkable in vivo nano silver accelerating effects on treatment of *S. aureus* infected skin wounds with no obvious side effects in mice [145].

## 14. Importance of nanocomposite

Nanocomposites are characterized by High dispersion in aqueous medium, uniform distribution of the active component for a long time, Controlled drug release, Reduction of frequency of administration, improvement of stability, penetrate regions inaccessible to other delivery systems [146,147].

## 15. Current applications of nanoparticles used in veterinary medicine

### 15.1. Medical diagnostics

Nanoparticles that used in this sector facilitate diagnosis and prognosis of diseases, availability for small samples and fast analyses of samples. But they have some disadvantages like difficult preparation of sample. Higher Sensitivity of small samples and price [148].

### 15.2. Biocides

They are alternatives to antibiotics and serve as antimicrobial coatings but they are cytotoxic and applied mainly in vitro [149,150].

## 16. Future perspective on the use of nanoparticles in veterinary medicine

As nanotechnology keeps on creating and collect more consideration, its applications in the creature generation industry will turn out to be increasingly broad. The customary consideration of nano-enhancements to strengthen animals feed is likely conceivable sooner rather than later; in any case, it will take more time for nanoparticles to completely supplant anti-infection agents in feed. The same number of biocidal competitors should in any case be tried in vivo before experiencing clinical preliminaries and sanitation tests as per government guidelines. For concentrates keen on nanoparticles with hostile to malignancy properties, it is critical to examine nanoparticle cytotoxicity in both disease cell lines and typical, solid cell lines. Just utilizing disease cells and guaranteeing the nano-molecule under scrutiny has hostile to malignancy properties might be deluding, as the nanoparticle might be cytotoxic to all cell types. In vivo examinations are required for verification of nanoparticle capacities seen in vitro research. Nonetheless, the advancement of perfect nanomaterials that are equipped for sending sign to the ailing or harmed cells and tissues to trigger the recovery procedure still stays a test. Also, the wellbeing of creatures as far as the utilization of nanomaterials in regenerative drug involves significant concern, since this field is still in its incipient stage. Employments of veterinary animals are progressively perceived as basic translational models of human illnesses. At long last, to comprehend the hidden components of cell-biomaterial associations at the nanoscale level, and to have the option to decipher the discoveries from seat to bedside, close cooperation between the researchers and veterinary clinicians is of most extreme significance. At long last, progresses in veterinary regenerative prescription could offer significant hotspots for contemplating human illness and furthermore it could offers a setting for dispersal of data about veterinary therapeutics that can quicken translational medication. Besides, the investigations are basic in the creature generation industry and especially to recognize the holes among learning and applications [151].

### 16.1. Examples of future applications of nanoparticles used in veterinary medicine

#### 16.1.1. Nutraceuticals

Nanoparticles used in this sector have many advantages such as; higher bioavailability of nutrients, enhancement of growth rate and performance, the most common limitation is the degradation within the gastrointestinal tract [151,152].

#### 16.1.2. Drug delivery systems

Used for enhancement of drug specificity and delivery. They diminish MIC and have a bactericidal effect on antibiotic-resistant strains of pathogens. Disadvantage is less biocompatibility [153,154].

#### 16.1.3. Sperm nanopurification

Depending on biomarkers selection and isolation of desired healthy sperm is performed. Nanoparticles used in this sector potentiate fertilization efficiency that facilitates fertilization of more females from a single collection. It had some limitation such as; to be developed it needs biomarker library and restrictions of purebred on artificial insemination [155].

## 17. Conclusion

In this paper, we reviewed different types of nanoparticles and different methods for their preparation and characterization, in addition, we reviewed the role of nanosystem in drug delivery of antimicrobials used in Veterinary Medicine with special consideration to silver and chitosan. Our willing study will be proposed to increase the efficacy of the selected antibiotics against pathogenic strains of bacteria, overcome the antibiotic resistance, decrease the toxicity and improve the bioavailability of selected antibiotic. The suggested protocol of the study is preparation of selected antibiotics loaded chitosan and silver nanoparticles characterization by this methods (XRD, Raman, FTIR, AFM, TEM, SEM, zeta seize and potential) and performing in-vitro studies including comparative efficacy of the tested drug and the prepared nanoparticles against standard, sensitive and resistant strains of micro-organism using different techniques as (sensitivity and MIC). According to the results of in-vitro studies, we will do the in-vivo studies which includes; comparative efficacy study of the tested drugs and the prepared nanoparticles in infected animals such as: *Pasteurella* infection in rabbit, *E-coli* infection in mice, infected wound in rat, comparative pharmacokinetic studies, comparative safety and toxicity studies of the tested drugs and the prepared nanoparticles using animal model. Investigation of anti-inflammatory effect of the tested nano drug after adding chitosan as it is known to possess anti-inflammatory activity.
